# Case Report: Uncommon presentation of *Schistosoma haematobium* infection in a migrant patient: diagnostic and therapeutic challenges

**DOI:** 10.3389/fmed.2025.1583233

**Published:** 2025-06-17

**Authors:** Francesca Carraturo, Maria Escolino, Assunta Russo, Angela Spagnuolo, Giovanni Esposito, Claudia Di Mento, Roberta Colicchio, Paola Salvatore, Ciro Esposito, Mariateresa Vitiello

**Affiliations:** ^1^Division of Pediatric Surgery, “Federico II” University Hospital, Naples, Italy; ^2^Department of Molecular Medicine and Medical Biotechnology, University of Naples “Federico II”, Naples, Italy; ^3^CEINGE-Advanced Biotechnologies Franco Salvatore s.c.ar.l., Naples, Italy

**Keywords:** schistosomiasis, hematuria, urinary bladder lesions, cystoscopic examination, Praziquantel

## Abstract

Schistosomiasis is a parasitic infection prevalent mainly in tropical and subtropical countries. We report a case of urinary infection caused by *Schistosoma haematobium* in a 16 years-old boy from Mali presented to our Institution with a one-month history of gross hematuria. The physical examination revealed an apparently healthy patient with soft abdomen, treatable, but painful to palpation and otherwise no other significant symptoms. The blood tests were within normal range except for eosinophilia. Urinary ultrasound revealed mild bilateral renal pelvis dilation, and distended bladder with thickened walls and multiple papillomatous growths (up to 31 mm in diameter). Cystoscopy confirmed multiple widespread mucosal lesions, and histology revealed severe eosinophilic cystitis with numerous parasitic eggs, observed, also, in parasitological urine examination, confirming the diagnosis of *S. haematobium* disease. The patient was diagnosed with schistosomiasis-related cystitis and treated with Praziquantel. At 2 months post-treatment follow-up urine microscope and eosinophil count were normalized and bladder wall irregularities and focal thickenings of variable size (from 3 to 11 mm) were documented. This case highlights the importance of considering hematuria and urinary bladder lesions in patients from areas where *Schistosoma* spp. is endemic as a strong indicator of parasitosis. Promptly initiating therapy can help prevent potential severe and less manageable consequences.

## 1 Introduction

Human schistosomiasis (1, 2), a neglected tropical disease, caused by helminth parasites from the *Schistosoma* genus, affects over 200 million people worldwide, primarily in Africa, Asia, and Latin America representing a major public health issue in tropical and subtropical countries (3–5). Europe is not generally endemic for human schistosomiasis, but it is affected by the disease in multiple ways. Environmental factors, such as water resource development, climate change, intensification of anthropogenic activities, increased migration flows from schistosomiasis-endemic regions, and the prolonged survival of adult parasites within the human host, potentially up to 40 years, may facilitate the emergence of autochthonous schistosomiasis, particularly in the southern regions of Europe (6–9).

Since 2014, numerous cases of acquired urogenital schistosomiasis have been described in Corsica (10, 11) where the parasite was introduced from an endemic region and established in local freshwater snails. In Italy, several studies have been performed on the prevalence of schistosomiasis in African migrants, showing a high incidence in this population. In 2019–2020, a study conducted by the National Institute for Health, found a prevalence of 31% for schistosoma infection, particularly in migrants from West Africa (12). Notably, the chronic urogenital manifestations of *S. haematobium* infection including hematuria, bladder wall thickening, and mass-like lesions can closely mimic neoplastic conditions, both clinically and radiologically. This diagnostic overlap often leads to delays or misdiagnosis, underscoring the importance of considering parasitic infections in the differential diagnosis of bladder cancers, particularly in individuals from endemic regions (1, 13, 14).

Six species of schistosomes infect humans: *S. haematobium*, *S. mansoni*, *S. japonicum*, *S. mekongi*, *S. intercalatum*, and *S. guineensis*. Among these, *S. haematobium* causes urogenital schistosomiasis. Infection occurs through contact with water containing schistosome cercariae, often during recreational, occupational, agricultural, or daily activities. Adult *S. haematobium* worms are localized in the retrovesical and periprostatic venous plexuses, depositing their eggs in the surrounding tissues, primarily in the bladder wall and urinary tract. These eggs cause inflammation and microlesions, resulting in hematuria, chronic cystitis, and, in severe cases, urinary obstructions. Each egg develops miracidium within a week, causing a local tissue reaction that facilitates its movement through the bladder layers into the lumen (13, 14).

Schistosomes have a complex life cycle, characterized of a sexual and asexual reproductive stage, with snails, specifically the *Bulinus truncatus* snail, as intermediate hosts and humans as the definitive hosts. Infected individuals excrete the parasite’s eggs in their stool and urine, which can contaminate both fresh and warm water, particularly in areas with inadequate sanitation. Once the eggs hatch, they release miracidia that infect the snails, where they reproduce asexually and eventually emerge as cercariae. These cercariae can penetrate human skin and transform into schistosomules. The young schistosomules move into the venous or lymphatic system, passing through the heart and lungs to the portal vein, where they mature within a few weeks. The female is transported by the male into the pelvic veins, completing its life cycle (13).

The most concerning outcome of urogenital schistosomiasis is the development of bladder cancer (14–16). Consequently, *S. haematobium* is classified as a biological carcinogen by the International Agency for Research on Cancer. Bladder cancer associated with chronic schistosomiasis involves immune dysregulation, where modulation of NK cell receptors plays a dual role in promoting anti-tumor activity while preserving healthy tissue integrity; in this context, tyrosine kinase inhibitors emerge as promising agents capable of targeting oncogenic signaling pathways while potentially influencing NK cell-mediated immune responses (17, 18).

Currently, the epidemiology is still poorly understood and due to gaps in surveillance and reporting.

Moreover, *S. haematobium* infection is often missed in early diagnoses due to symptom overlap with other urinary diseases, yet timely detection and treatment are crucial to prevent long-term complications. Reducing the risk of late diagnosis is essential, especially as this insidious and neglected disease has recently re-emerged in Europe (9). This issue is particularly relevant among migrant populations, who are affected by structural barriers that compromise timely access to medical care, such as language discordance and the lack of accessible or reliable medical history documentation. Diagnostic delays in such contexts may lead to prolonged morbidity, missed opportunities for early therapeutic intervention, and an elevated risk of adverse outcomes, including irreversible organ damage or malignant transformation. Addressing these gaps is essential to ensure equitable healthcare delivery and to improve clinical outcomes in at-risk and vulnerable populations.

Here, clinical and laboratory approaches were used to investigate a case of *S. haematobium* infection in a migrant boy from Mali, aiming to confirm diagnosis and assess response to Praziquantel (PZQ) therapy. Case outcomes and implications are discussed.

## 2 Case report

### 2.1 Clinical presentation

A 16 years-old patient from Mali was referred to our Institution complaining of gross hematuria since June 2023, without fever, chills, vomiting, diarrhea, and other symptoms. He was apparently healthy, although limited available medical and family history due to language barriers and a lack of cooperation (currently under the care of a family home guardian). Following the appearance of hematuria, a standard urine test and an ultrasound of the upper and lower abdomen were ordered at an outside facility.

On 6 June 2023, the urinalysis showed hemoglobin, numerous red blood cells, and leukocytes. An abdomen ultrasound conducted on 31 June 2023, revealed no evidence of abdominal fluid accumulation, mild bilateral dilation of the renal pelvis, a distended bladder with diffusely thickened walls, and irregular contours caused by multiple papillomatous growths (up to 16 mm in diameter) accompanied by mobile echogenic material in the dependent areas, and a non-uniform prostate structure. No signs of parenchymatous or cholestatic liver injury, or advanced liver pathology were observed.

The patient was referred for urological and cystoscopic evaluation and admitted to our Pediatric Surgery Unit. At the time of admission, the patient’s overall clinical condition was good, except for hematuria episodes over the past month and the presence of a reducible umbilical hernia.

### 2.2 Differential diagnoses, diagnostic workup and therapeutic interventions

During hospitalization in the Pediatric Surgery Unit, between 28*^th^* August and 30*^th^* August, the patient underwent laboratory tests (Table 1). In a young patient from a schistosomiasis-endemic area, presenting hematuria and bladder wall lesions, the differential diagnosis is broad and requires a multidisciplinary approach. One of the initial concerns was the possibility of bladder neoplasia. The presence of papillomatous growths on imaging and cystoscopy, along with hematuria, could raise suspicion for this malignancy. Another important differential diagnosis was urinary tract tuberculosis (TB), particularly due to the granulomatous features observed in the bladder biopsies. TB can affect the genitourinary system, and its presentation may mimic neoplastic or inflammatory bladder disease. However, extensive testing including Interferon Gamma Release Assay (LIAISON^®^ QuantiFERON^®^ TB-Gold Plus, Diasorin) with values of 0,012 IU/mL (TB1-CD4^+^) and 0,082 IU/mL (TB2-CD4^+^/CD8^+^), *Mycobacterium tuberculosis*-polymerase chain reaction of urine and Mantoux tuberculin skin test were performed and were consistently negative, making this diagnosis unlikely. The hypothesis of eosinophilic cystitis also emerged early in the diagnostic workup, especially after histological analysis of bladder biopsies revealed dense eosinophilic infiltration and granulomatous inflammation. Eosinophilic cystitis is a rare inflammatory condition that can clinically and radiologically mimic bladder cancer. It is often associated with allergies, infections, or parasitic infestations and thus served as a valuable diagnostic clue pointing toward a potential helminthic etiology. Chronic bacterial cystitis was also considered, as it can cause hematuria and bladder wall thickening. However, repeated urine cultures did not reveal any pathogenic bacterial growth, and there was no evidence of nitrites in urinalysis or systemic signs of infection. Enterobacteriaceae and enteropathogens (*Salmonella*, *Shigella*, *Yersinia*, *Campylobacter*, *Escherichia coli* O157H7) were also negative in the extended stool-culture on Xylose lysine desoxycholate agar (XLD; Difco, Sparks, MD).

**TABLE 1:** Results of laboratory workup.

Microbiological tests	Normal value	Test results
Interferon gamma release assay	< 0.35 IU/mL: Negative	0.012 IU/mL (TB1-CD4^+^) 0.082 IU/mL (TB2-CD4^+^/CD8^+^)
*Mycobacterium tubercolosis*-polimerase chain reaction of urine	Negative	Negative
Mantoux tuberculin skin test	Negative	Negative
Anti-*Strongyloides stercoralis* antibodies with ELISA test	< 9 NTU: Negative	7 NTU
Stool extended bacterial pathogens culture (*Salmonella, Shigella, Yersinia, Campylobacter, Escherichia coli O157H7*)	Negative	Negative
Complete blood count	Normal value	Test results
White blood cell count	4.8–10.8 × 10^3^/mL	9.74 × 10^3^/mL
Lymphocytes	1.0–4.8 × 10^3^/mL	2.80 × 10^3^/mL
Monocytes	0.1–0.8 × 10^3^/mL	0.51 × 10^3^/mL
Eosinophiles	0.0–0.45 × 10^3^/mL	2.78 × 10^3^/mL
Basophils	0.0–0.20 × 10^3^/mL	0.10 × 10^3^/mL
Red blood cell count	4.2–5.6 × 10^3^/mL	5.72 × 10^3^/mL
Hemoglobin	12–17.5 g/dL	15.4 g/dL
Platelet count	130–400 × 10^3^/mL	207 × 10^3^/mL
Inflammatory markers	Normal value	Test results
C-reactive protein	0–5 mg/dL	3.4 mg/dL
Fibrinogen	160–350 mg/dL	353 mg/dL
Albumin	3.8–5.4 g/L	4.3 g/L
Total protein	6–8 g/dL	8 g/dL
Urine examination	Normal value	Test results
Erythrocytes	0–14 cells/μL	316 cells/μL
Leukocytes	0–18 cells/μL	382 cells/μL
pH	5–9	6.5
Leukocyte esterase	Absent	Revealed in traces
Nitrites	Absent	Absent
Urine bacteriological test	Negative	Negative
Urine parasitological test	Negative	Presence of *Schistosoma hematobium* eggs

Given the geographical origin of the patient, parasitic infections should be considered predominant in the differential diagnosis. Moreover, no anti-*Strongyloides stercoralis* antibodies were detected in the patient’s serum using an ELISA method (The NovaLisa^®^ Strongyloides Antibody ELISA, Novatec Immunodiagnostic GmbH) that resulted with a negative value (the diagnostic specificity of this assay is 94.12% (95% confidence interval: 83.76%–98.77%), and the diagnostic sensitivity is 89.47% (95% confidence interval: 75.2%–97.06%).

The complete blood count revealed a white blood cell count of 9.74 × 10^3^/mL (range 4.8–10.8 × 10^3^/mL), hemoglobin 15.4 g/dL (range 12–17.5 g/dL), and platelets 207 × 10^3^/mL (range 130–400 × 10^3^/mL). The differential white blood cell count was normal, except for mild eosinophilia, with 2.78 × 10^3^/mL (range 0.0–0.45 × 10^3^/mL). The blood chemistry levels were all within normal ranges with normal inflammatory markers.

Physical-chemical urine examination showed in the urine sediment the presence of numerous erythrocytes, 316 cells/μl (range 0–14) and leukocytes, 382 cells/μl (range 0–18) associated with traces of leukocyte esterase.

The urinary ultrasound, performed on 28 August 2023, showed normal-sized kidneys with regular margins in their anatomic position. The parenchymal thickness was at the lower limit of normal, and the corticomedullary ratio was appropriate for the patient’s age. Mild hyperechogenicity was noted in the right kidney. No signs of obstructive pathology were observed. The bladder was distended with finely and diffusely thickened walls, showing evidence of diffuse endoluminal sediment. At least six large, nodular, vegetating formations, adherent to the bladder walls, were visible with hyperechoic structures. These were located as follows: - in the left angular basal region (approximately 11 × 15 mm), - two along the left lateral wall (10 × 7 and 15 × 10 mm), - along the right lateral wall (11 × 5 mm), and the two largest on the bladder dome, protruding into the lumen and extending along the right lateral wall, with associated hypoechoic components (26 × 19 mm); the final formation (maximal diameters 31 × 11 mm) wrapped around the right ureter, which appeared ectatic at its distal segment. No free fluid is present in the pelvic cavity.

Further investigation was necessary, including chest X-ray and cystoscopic examination. The chest X-ray of 29^th^ August showed mild reinforcement of the vascular-bronchial markings, particularly in the hiloperibronchial and hilar-basal regions. The diaphragm appeared normal in profile, with clear costophrenic angles. Cardiac volume was within normal limits. Cystoscopic examination revealed multiple papillomatous formations adherent to the bladder walls, with a ubiquitous distribution.

**FIGURE 1:**
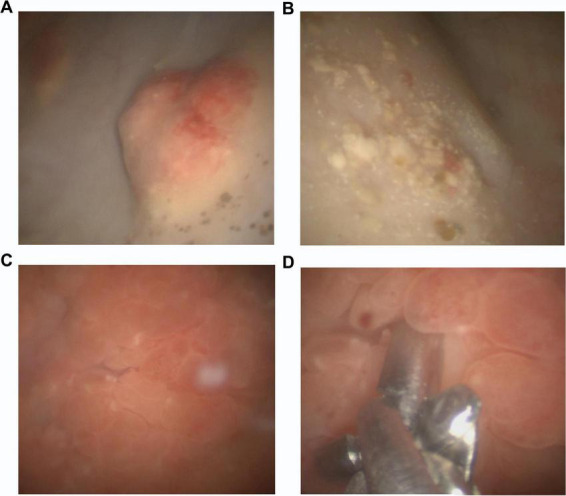
Cystoscopic images. **(A,B)** “Sandy patches” and mucosal nodularity with erythema and hemorrhage, **(C,D)** multiple and inflamed papillomatous lesions.

A critical diagnostic pitfall is the visual similarity between schistosomal granulomas and papillomatous tumors. This underlines the importance of systematic histological sampling and urinary cytology in all cases of persistent or atypical bladder lesions. As a result, multiple biopsies of these formations were performed (Figure 1). Histological analysis showed a pattern of eosinophilic cystitis with granulomatous features. This is consistent with an inactive stage of schistosomiasis.

**FIGURE 2:**
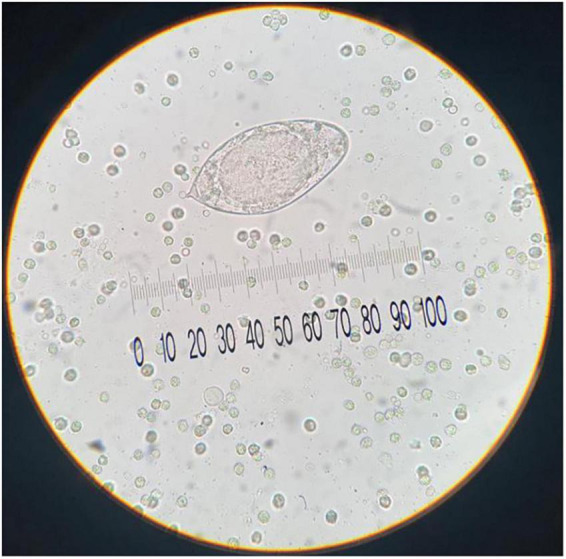
Urine sediment examination shows a representative egg of *S. haematobium* with diagnostic sub-terminal spine (approximative size: 150 μm; original magnification, 40x; no stain used).

In addition to the abnormalities observed in urinalysis and instrumental investigations, a microscopic parasitological examination of urine was also critical. The microscopic observation, as shown in Figure 2, revealed the presence of eggs of approximately above 150 μm in size, embryonated, with elongated or ellipsoid shape and a sub-terminal spine that were morphologically compatible with *S. haematobium* eggs. Following the detection of *S. haematobium* in the urine parasitology test, the patient was transferred to the Pediatric Infectious Diseases Department on 01/09. The team there noted that the patient was in good overall clinical condition. The brain CT scan showed no significant abnormalities, and the neurological exam was unremarkable. Blood tests once again revealed eosinophilia, strongly indicative of a parasitic infection.

The patient was treated with 600 mg of PZQ (Chandra Bhagat Pharma Ltd) (20 mg/kg in three administrations for 1 day only). After the treatment with PZQ, the patient did not experience any further episodes of hematuria and was discharged once the stability of their general condition was confirmed.

Every month, the patient underwent Day Hospital treatment in the Pediatric Infectious Diseases Department for a complete blood count, PCR, biochemistry tests, and especially for the urine collection to search for Schistosoma. Despite persistent negative urine parasitology and normalized eosinophil counts during follow-up, bladder wall irregularities and focal thickenings of variable size (from 3 to 11 mm) were documented up to date, demonstrating that PZQ was effective. No evidence of malignancy was found in repeated cytological examinations.

## 3 Discussion

Schistosomiasis is a critical public health concern, especially in its urinary form (14–16, 19). This condition mainly results from the long-term impacts of the infection rather than from the acute phase, which is manageable with treatment. The ongoing presence of the disease in endemic areas is complicated by education gaps and local environmental factors. However, the number of newly diagnosed cases of schistosomiasis is increasing in non-endemic areas, such as the Mediterranean regions of Europe, due to international tourism and immigration from transmission areas (6–9, 20).

Although 40% of infected subjects are asymptomatic, specific signs are present in 24%–66.4% of cases. Clinical outcomes are generally mild, but severe and unusual forms of the disease may occur. Severe infections are often associated with increased clinical severity due to allergic host responses, although significant morbidity may also occur from mild or mild infections. Chronic conditions arise from granulomatous inflammation due to egg deposition in tissues, with dysuria and hematuria being common symptoms at all stages of the disease. Late-stage complications can include proteinuria (often nephrotic), bladder calcifications, chronic bladder ulceration, ureter obstruction, renal colic, hydronephrosis, renal failure, and, ultimately, bladder carcinoma (14–16).

However, *Schistosoma* spp. infections can be asymptomatic, with no clinical signs or eosinophilia present (1, 2, 21, 22). As a result, schistosomiasis may be misdiagnosed and lead to high severity and chronicity with increased disability.

Therefore, laboratory diagnosis is crucial to accurately identify schistosomiasis. The diagnosis of schistosomiasis requires confirming infection, but the sensitivity of the available tests’ ranges significantly, from 45% to 72%. This includes serological assays for schistosomes antibody and microscopic detection of eggs in urine or stool (2, 21, 22).

Serologic assays for *Schistosoma* spp. can detect infection earlier than microscopic examination but cannot differentiate between active and prior infections, remaining positive for life. They are most useful in non-immune individuals, such as returning travelers with recent exposure. Identification of the eggs in urine or stools by microscopy technique is the most practical diagnostic method, being the unit of measurement of the number of eggs per gram of urine or stools. For suspected *S. haematobium* infection, the urine test is preferred, and specimen collection should occur at least two months after the last exposure to allow time for adult worms to mature and produce eggs.

Urine microscopic examination in our patient revealed ova, with specific size, shape, and spine characteristics to support *S. haematobium* identification.

Moreover, in hospital settings, cystoscopy and endoscopy are utilized to visualize lesions linked to urinary schistosomiasis. A common cystoscopy observation is the presence of “sandy patches” and mucosal nodularity. If the submucosa is involved, fibrosis or calcification may hinder bladder contraction, resulting in hypertonic or atonic bladder conditions (14–16). Radiological methods can reveal pathological lesions associated with *S. haematobium* infection, with calcifications in the ureter and bladder wall—due to calcified eggs in the submucosa—being the most frequent radiological finding in areas where the disease is common (23). Histopathological examination often reveals eosinophilic infiltration, fibrosis, and squamous metaplasia, with or without identifiable ova, complicating differentiation from malignancy (14–16). A multidisciplinary diagnostic approach, integrating parasitological, radiological, cytological, and histological findings, is essential for accurate diagnosis and appropriate management in patients from endemic regions.

Our patient presented microscopic and macroscopic hematuria associated with moderate eosinophilia, which suggested a helminthic infection. The subsequent investigation by urinary ultrasound and cystoscopy observation revealed the finding of the multiple papillomatous growths, which were resected. Histopathological evaluations consistently demonstrated eosinophilic cystitis with granulomatous inflammation, urothelial metaplasia, and calcified *Schistosoma* ova. The presence of bladder lesions and urothelial metaplasia raised concern for potential neoplastic transformation. However, repeated negative cytology, absence of dysplasia in biopsies, and stable clinical status argue against malignancy.

Therefore, the key strategies for managing schistosomiasis should include early diagnosis, intensive treatment, and regular follow-up care.

In response to the diagnostic challenges associated with hematuria and bladder lesions, particularly the difficulty in differentiating *S. haematobium* infection from neoplasia, we recommend a diagnostic algorithm based on the patient’s epidemiological setting. In endemic areas, the initial screening should emphasize non-invasive methods, including clinical assessment focused on urogenital symptoms (gross/microscopic hematuria, dysuria), urine microscopy for detection of schistosome eggs and molecular diagnostics assays for increased sensitivity in low-intensity *S. haematobium* infections. If results are inconclusive or there is persistent clinical suspicion of neoplasia, further investigation with cystoscopy and histopathological biopsy is appropriate. In non-endemic regions, detailed travel and migration history is crucial. The diagnostic approach should include serological testing for anti-schistosome antibodies used for screening, urine sediment examination and molecular assays before progressing to invasive procedures. Radiological imaging (ultrasound or CT urography) can provide additional information on the morphology and extent of bladder lesions. This stratified, epidemiology-based, approach optimizes diagnostic accuracy while minimizing unnecessary invasive techniques, and facilitates early detection and management of urogenital schistosomiasis, particularly in cases where delayed diagnosis may lead to complications such as bladder fibrosis, calcifications, or squamous cell carcinoma (1).

Praziquantel, a pyrazinoisoquinoline derivative, is currently the only drug available for the treatment of all *Schistosoma* spp. It is typically administered as a single dose of 40 mg/kg body weight for both school-aged children and adults through mass drug administration programs. However, its effectiveness ranges between 70% and 80% in adults and schoolchildren, with lower efficacy in preschool-aged children. Contributing factors to reduced effectiveness in young children include inadequate dosing, poor adherence due to the drug’s unpleasant taste, and limited understanding of PZQ’s pharmacokinetics and pharmacodynamics in this age group (2, 24).

Extended post-treatment follow-up to 18 months, monitoring of eosinophil level, parasitological urine examination, and clinical/imaging evaluation revealed that PZQ treatment was likely successful. The patient experienced no further episodes of hematuria and was discharged once his general condition stabilized.

## 4 Conclusion

Schistosomiasis is an emerging health issue in non-endemic areas, largely due to migration. Often misdiagnosed, it can lead to serious complications if not promptly addressed.

This report details a case involving a young boy from Mali with hematuria and a bladder lesion linked to *S. haematobium*. A combination of parasitological tests, imaging, and tissue analysis enabled early intervention, preventing severe complications like bladder cancer and renal failure.

From a clinical practice perspective in non-endemic regions, this case emphasizes the importance of considering parasitic infections in the differential diagnosis of hematuria and bladder lesions, especially in migrant populations. With rising global mobility, awareness and swift recognition of such infections are crucial to avoid delayed care and long-term damage.

## Data Availability

The raw data supporting the conclusions of this article will be made available by the authors, without undue reservation.
